# Inflammatory markers as potential mediators on the negative association between training load and bone mineral density in adolescent competitive swimmers: ABCD-growth study

**DOI:** 10.3389/fendo.2025.1602551

**Published:** 2025-08-14

**Authors:** Ricardo R. Agostinete, Pedro H. Narciso, Manuel João Coelho-e-Silva, Renata M. Bielemann, Luis Alberto Gobbo, Bruna Camilo Turi-Lynch, Romulo Araújo Fernandes, Dimitris Vlachopoulos

**Affiliations:** ^1^ Laboratory of Investigation in Exercise (LIVE), Department of Physical Education, Sao Paulo State University (UNESP), Presidente Prudente, Brazil; ^2^ CIDAF (UID/DTP/04213/2016), Faculty of Sport Sciences and Physical Education, University of Coimbra, Coimbra, Portugal; ^3^ Post-Graduate Program in Nutrition and Foods, Federal University of Pelotas, Pelotas, Brazil. Post-Graduate Program in Epidemiology, Federal University of Pelotas, Pelotas, Brazil; ^4^ Skeletal Muscle Assessment Laboratory (LABSIM), Department of Physical Education, School of Technology and Sciences, São Paulo State University (UNESP), Presidente Prudente, Brazil; ^5^ Department of Physical Education and Exercise Science, Lander University, Greenwood, SC, United States; ^6^ Children’s Health and Exercise Research Centre, Sport and Health Sciences, University of Exeter, Exeter, United Kingdom

**Keywords:** youth, athletes, bone health, cytokines, hormones

## Abstract

**Introduction:**

Competitive swimming during adolescence has been linked to poor bone development, potentially influenced by training load, inflammation, hormones, and bone markers. However, this influence has been poorly investigated in the literature.

**Objective:**

To compare whether competitive adolescent swimmers present differences in inflammatory, immunological, anabolic, and bone markers compared with non-sport group and to analyse whether inflammatory variables mediate the association between training load and areal bone mineral density (aBMD) in the swimmers group.

**Methods:**

This cross-sectional study included 61 adolescents (20 females, 15.4±2.3 years), of which 30 were adolescent swimmers and 31 did not participate in sports (non-sports group). The daily training load was obtained by multiplying the perceived exertion score by the training volume of the swimmers. Lean soft tissue, fat mass and aBMD were estimated from whole-body scans using dual-energy X-ray absorptiometry and peak height velocity considering stature and body mass. Blood samples were collected to assess bone markers (calcium, 25-hydroxy vitamin D, C-terminal telopeptide, and osteocalcin), growth hormones, insulin-like growth factor 1 (IGF-1), lymphocytes, leukocytes, and inflammatory markers (interleukin-6 [IL-6] and C-reactive protein [CRP]). Statistical analyses applied a significance level at p<0.05.

**Results:**

Besides lower values in BMD (expect in upper limbs), swimmers had higher calcium (10.0 ± 0.30 vs 9.7 ± 0.44, p=0.007), vitamin D (42.6 ± 10.4 vs 24.2 ± 5.9, p<0.001), and IGF-1 (397.2 ± 115.1 vs 220.3± 73.9, p<0.001) concentrations than their non-sports peers. The mediation analysis found no indirect associations between training load and aBMD through inflammatory markers. Nevertheless, training load was directly and negatively associated with aBMD in the lower limbs (β=-0.1533, 95%CI:-0.2875, -0.0191) and total body less head (β=-0.0978, 95%CI:-0.1880, -0.0076) through IL-6 and directly and negatively associated with aBMD at all sites through CRP.

**Conclusion:**

The swimming and non-sports groups did not show differences in bone, inflammatory, or immunological markers. In contrast, swimmers had higher concentrations of IGF-1, calcium, and 25-hydroxy vitamin D. Although the training load was negatively associated with aBMD, inflammatory markers (IL-6 and CRP) did not mediate this association. Reinforcing the hypothesis that swimmers have lower aBMD due to the hypogravitational environment.

## 1 Introduction

The literature shows that sports with different mechanical impacts can distinctly affect the bone mineral density (BMD) (1). Sports with a higher mechanical impact and muscle activity, i.e. basketball and gymnastic, seem to be more effective for areal bone mineral density (aBMD) accrual (2), mainly during adolescence, when most of the bone mass observed in adulthood is accumulated (3). In addition, sports without a mechanical impact, such as swimming, are not beneficial for bone health, even with high muscle activity (4). Recent studies have shown that swimming can similarly or negatively affect the bone health of adolescents, even when compared to adolescents not engaged in sports, as observed in lower limbs and whole-body aBMD (4, 5).

Therefore, it is important to understand whether other factors, such as inflammation, could explain the negative association between training load and aBMD, in addition to hypogravity. Evidence indicates that the training load and previous sports practice time are negatively correlated with aBMD in adolescent swimmers (6, 7). Furthermore, training with very high physical effort (e.g., ~74% VO_2max_ or >64% VO_2max_) may favour the increased circulation of immunological variables (8, 9), cytokines, and proteins related to the inflammatory response, such as interleukin-6 (IL-6), interleukin-1 (IL-1), tumour necrosis factor alpha (TNF-α), and C-reactive protein (CRP) (10). However, an intense bout of exercise in adolescents can lead to an acute reduction of anabolic markers such as insulin-like growth factor 1 (IGF-1) immediately after exercise (11), which is important for bone growth (12). Among the pro-inflammatory markers, IL-6 cytokine deserves special attention due to its increased expression on the activator of nuclear factor-kB ligand (RANKL), an essential protein for osteoclastogenesis (bone resorption) (13, 14). The increase in this process resultant of osteoclast activity may reduce bone mass accrual, resulting in decreased BMD. Additionally, evidence indicates that IL-6 leads to the increased production of CRP (15), which is a marker of inflammation in clinical practice (16).

However, to our knowledge, no studies have analysed whether, in fact, a competitive swimming training routine can cause changes in inflammatory, immunological, anabolic, and bone markers in adolescents compared with a non-sports group. Furthermore, whether inflammatory markers mediate the negative association between training load and BMD in swimmers has not yet been analysed. We hypothesized that adolescent competitive swimmers exhibit differences in physiological markers compared to controls, with inflammatory markers potentially mediating the negative association between training load and BMD. Thus, this cross-sectional study firstly aimed to compare whether competitive adolescent swimmers present differences in inflammatory, immunological, anabolic, and bone markers compared to adolescents who do not perform organised sports. Secondly, we analyzed whether inflammatory variables mediate the association between training load and aBMD in adolescent swimmers.

## 2 Methods

### 2.1 Study design

This was a cross-sectional study, and the data were collected as part of ongoing research, titled “Analysis of Behaviors of Children During Growth: ABCD-Growth Study”. The data from this study were collected as part of investigations by the Laboratory of InVestigation in Exercise (LIVE) conducted in Presidente Prudente, São Paulo, Brazil, from 2017 to 2018. The ABCD Growth Study aims to identify the impact of physical activity and sports participation on health variables, including bone tissue. This study was approved by the Ethical Research Committee of the São Paulo State University-UNESP (process number: 02891112.6.0000.5402).

### 2.2 Sample

The sports group comprised adolescents from a swimming team classified in Tier 2: Trained/Developmental, from Participant Classification Framework proposed by McKay et al. (2022) (17). While adolescents not engaged in organised sports were invited to join the non-sports group. To participate in the study, the adolescents were required to provide informed consent signed by their parents or guardians in according with the Declaration of Helsinki.

For adolescents who participated in sports, the following inclusion criteria were adopted: i) previous involvement for at least six months in the current sport (in the present study sample, the minium period of engagement observed in swimming was 24 months), ii) signed written consent form, iii) absence of clinical or metabolic disorders (previously diagnosed), iv) no regular use of medications that may affect the development and growth of the adolescent, and v) age of 10–19 years. For the adolescents in the non-sports group, the following criteria were applied: i) no regular participation in sports, ii) signed written consent, iii) absence of clinical or metabolic disorders (previously diagnosed), iv) no regular use of medications, and iv) age of 10–19 years.

The sample size was estimated based on the study by Agostinete et al. (6), using a correlation (two tailed bivariate correlation) coefficient of R=0.563 between the training load and lower limb aBMD, an alpha error of 0.05, a statistical power of 80%. The minimum estimated sample size was 22 participants, which was calculated using G*Power software. Thus, the final sample comprised 61 adolescents (41 males and 20 females) divided into two groups: 30 adolescent swimmers (21 males and 9 females) and 31 adolescents in the non-sports group (20 males and 11 females).

### 2.3 Outcomes

Height was measured using a fixed stadiometer (accurate to 0.1 cm; Sanny model, American Medical Do Brazil Ltda, Brazil), and body mass was measured using an electronic scale (accurate to 0.1 kg; Filizola PL 150 model; Filizzola Ltda, Brazil).

Biological maturation was estimated by the maturity offset (MO) using mathematical models based on anthropometric measures (18) for males = -7.999994+ [0.0036124 * (Age * Stature)] and females = -7.709133+ [0.0036124 * (Age * Stature)]. Thus, peak height velocity (PHV), the period of age where the fastest growth is seen, is given by the difference between chronological age and maturity offset (PHV = chronological age – MO).

To assess the whole-body aBMD (g/cm²), lean soft tissue (kg), and fat mass (kg and %), dual-energy x-ray absorptiometry (DXA) scanning (models Lunar DPX-NT and Lunar Prodigy advance; General Electric Healthcare, Little Chalfont, Buckinghamshire, UK) was performed at the university laboratory in a temperature-controlled room using GE Medical System Lunar software. Each day, a trained researcher performed the scans and tested the scanner quality before the first examination. The scans were performed using a standardised protocol, with the participants remaining in the supine position and wearing light clothing without shoes. Regional BMD analysis according to the definitions of the regions of interest (ROIs), including the upper limbs, lower limbs, spine, and whole body (less the head), occurred offline after the scans were performed, as previously described (19).

Blood samples were collected by a certified nurse. Adolescents were instructed to refrain from exercise training on the day previous to blood collection. After an overnight fast, venous blood was collected from the antecubital vein in vacuum collection tubes with anticoagulant separating gel. Blood collection was carried out in a private laboratory, where it was also analyzed, and all the quality control standards adopted by the Brazilian Health Ministry were followed. Bone turnover markers, such as calcium, 25-hydroxy vitamin D, C-terminal telopeptide (CTX), osteocalcin, growth hormone, insulin, and IGF-1, were analysed using an electrochemiluminescence kit (Roche), Cobas e170, and Modular Analytics E170 equipment (chemiluminescence assay kit [VITROS] with the VITROS^®^ XT 7600 equipment). Lymphocytes (CD3+, CD4+, and CD8+ cells) and leukocytes were analysed by flow cytometry using a Beckman Coulter kit and the Aquios-Beckman Coulter equipment. Finally, IL-6 and CRP levels were analysed using a Siemens kit and Immulite 2000 XPI equipment (Siemens Healthcare GmbH, Erlanger-Germany). Due to the sensitivity of the kit used, IL-6 values <1.5 were defined as 1.4 for the statistical analyses.

The training load was measured in the swimming group. The adolescents reported the intensity using the rating of perceived exertion proposed by Borg in 1982 and adapted by Foster (20) and the volume of training sessions (in hours and minutes) during the entire month (considering the days of training) prior to data collection. This method has been applied previously in studies involving adolescent swimmers, (6) and the daily training load was obtained by multiplying the rating of the perceived exertion score by the training volume (in minutes). The sum of the daily training loads was used to obtain the monthly training load used in this study.

### 2.4 Statistical analysis

**Figure 1:**
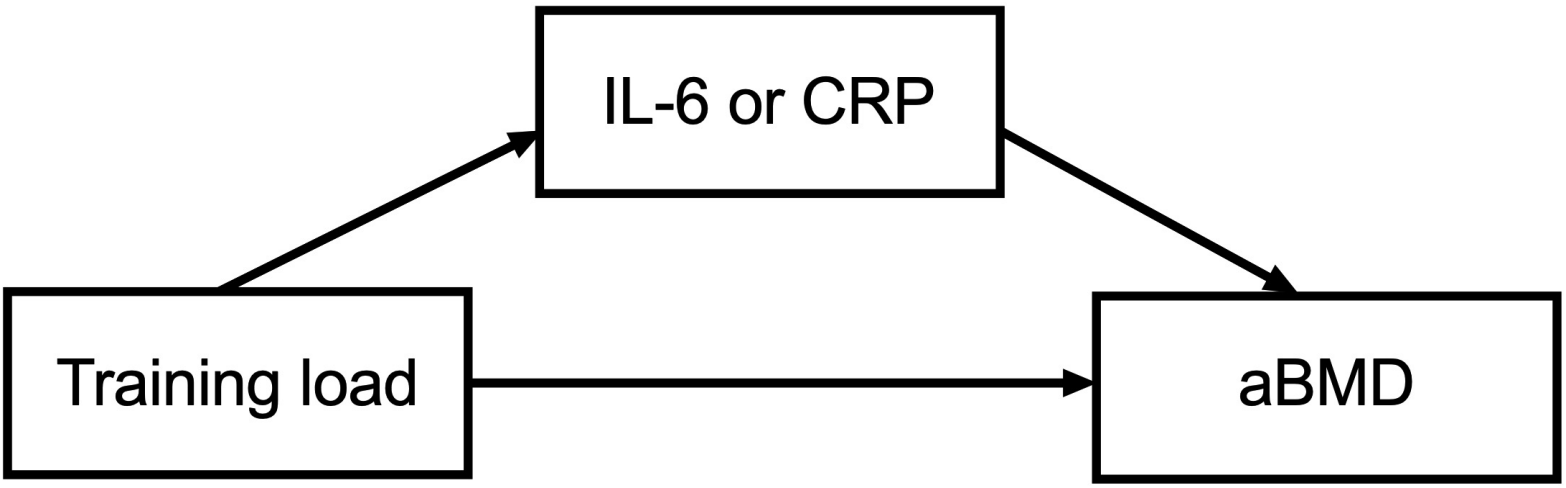
Theoretical framework of the mediation model. Independent variable: training load; dependent variable: areal bone mineral density (aBMD); mediator variables: inflammatory markers (IL-6 or CRP). Confounders: chronological age and APHV.

Descriptive statistics included means, standard deviations, and confidence intervals (95% CIs). A generalized estimating equation (GEE) was used to compare variables between groups by applying linear or gamma scale responses, when appropriate, and considering poshoc of Bonferroni. All comparisions were performed in SPSS (version 24.0). Since the adjusted GEE analysis do not provide standard deviations (SD), these values were calculated using the following formula: $SD=SEM\;x\sqrt{n}$ (21), where SEM was obtained from stardard error (std. error). In sequence, it was calculated the SD pooled applying the formula: $SD_{\text{pooled}}=\sqrt{\frac{\left(n_{1}-1\right)SD_{1}^{2}+\left(n_{2}-1\right)SD_{2}^{2}}{n_{1}+n_{2}-2}}$ . Lastly, the cohen’s d effect sizes were calculated following the equation: $d=\frac{{\text{mean}}_{1}-{\text{mean}}_{2}}{SD_{\text{pooled}}}$ . The magnitude of effect size was calculated considering the following cut-off points: trivial ≤ 0.20, small = 0.20–0.59, moderate = 0.60–1.19, large =1.20–1.99, and very large ≥ 2.00 (22). Mediation analyses were performed using the model proposed by Valeri and Vanderweele (23) and applying the “med4way” command in Stata (version 14.0) and the theorical model is presented in **Figure 1**. This method decomposed the total association between training load and aBMD into direct (i.e., the direct effect of training load on aBMD through pathways not related to inflammatory markers) and pure indirect (i.e. the effect of training load on aBMD mediated by inflammatory markers) effects. In addition, we analysed the reference (i.e. the effect of training load on aBMD due to interactions with inflammatory markers) and mediated (i.e. the effect of training load on aBMD due to both mediation by and interaction with inflammatory markers) interactions. All mediation analyses were adjusted for chronological age and APHV. For all analyses, the significance level was set at p<0.05.

## 3 Results

The characteristics of the participants are shown in **Table 1**. Swimmers and control groups presented similar values for all anthropometric, maturational, and body composition variables in crude comparisons, except for body mass (p=0.022), fat mass in kilograms (p=0.004), and fat mass percentage (p=0.040). **Table 2** presents the adjusted comparisons of aBMD, bone markers, and hormone/inflammatory variables between the swimmers and the control group. Swimmers showed lower aBMD in the lower limbs (p=0.011, d=0.59, small effect size), spine (p<0.001, d=1.09, moderate effect size), and total body less the head (TBLH; p<0.001, d=0.80, moderate effect size) than the control group. Regarding the bone markers, swimmers had higher calcium (p=0.002, d=0.74, moderate effect size) and vitamin D (p<0.001, d=2.18, very large effect size) concentrations. The same pattern was observed for hormonal variables such as IGF-1 (p<0.001, d=1.84, large effect size).

**Table 1:** Descriptive characteristics of the participants stratified by swimming participation (n=61).

Independent variables	Swimming group (n= 30)	Control group (n=31)	GEE
	Mean (SD)	Mean (SD)	P-value
General information			
Sex (Male/Female)	21/9	20/11	–
Chronological age (years)	15.0 (2.5)	15.9 (2.1)	0.132
MO (years)	1.5 (2.1)	2.3 (1.8)	0.547
Age at PHV (years)	13.5 (1.0)	13.6 (0.9)	0.699
Body mass (kg)	55.1 (11.9)	62.3 (13.3)	**0.022**
Stature (cm)	165.0 (10.4)	167.7 (10.3)	0.295
Lean soft tissue (kg)	42.7 (11.3)	43.9 (10.6)	0.661
Fat mass (kg)	10.5 (4.5)	15.5 (8.5)	**0.002**
Fat mass (%)	19.3 (8.0)	24.5 (11.3)	**0.032**
Training variables			
Engagement period (months)	68.00 (41.52)	–	–
Weekly frequence (days/week)	5.97 (0.18)		
Training volume (min/month)	1794.1 (583.1)	–	–
Training Intensity (RPE/month)	59.8 (18.7)	–	–
Training load (RPE x min)	6305.8 (2229.7)	–	–

SD, standard deviation; GEE, generalized estimating equation, MO, maturity offset; PHV, peak height velocity; RPE, Rating of perceived exertion. Significant results are presented in bold.

**Table 2:** Comparison of markers by swimming participation (n=61).

Independent variables	Swimming group (n=30) Mean ± SD (95%CI)	Non-Sports group (n=31) Mean ± SD (95%CI)	GEE-p	Cohen’s d
Bone mineral density #				
Upper Limbs (g/cm^2^)	0.828 ± 0.037 (0.815, 0.842)	0.839 ± 0.084 (0.810, 0.869)	0.501	0.17
Lower Limbs (g/cm^2^)	1.175 ± 0.075 (1.148, 1.202)	1.223 ± 0.089 (1.192, 1.255)	**0.026**	0.59
Total Spine (g/cm^2^)	0.941 ± 0.069 (0.916, 0.966)	1.042± 0.111 (1.004, 1.082)	**<0.001**	1.09
WBLH (g/cm^2^)	0.983 ± 0.047 (0.966, 1.000)	1.033 ± 0.076 (1.007, 1.060)	**0.002**	0.80
Bone markers ‡				
Calcium (mg/dL)	10.0 ± 0.30 (9.9, 10.1)	9.7± 0.44 (9.5, 9.8)	**0.007**	0.74
25-hydroxy vitamin D (ng/mL)*	42.6 ± 10.4 (39.0, 46.5)	24.2 ± 5.9 (22.2, 26.4)	**<0.001**	2.18
Bone turnover markers ‡				
CTX (ng/mL)	0.831 ± 0.270 (0.739, 0.933)	0.990 ± 0.394 (0.860, 1.139)	0.070	0.47
Osteocalcin	55.2 ± 23.4 (47.4, 64.2)	48.1 ± 28.8 (38.9, 59.3)	0.319	0.27
Hormones ‡				
Growth hormone (ng/mL)	2.42 ± 4.00 (1.34, 4.37)	1.24 ± 1.80 (0.74, 2.07)	0.123	0.38
IGF-1 (ng/mL)	397.2 ± 115.1 (358.0, 440.6)	220.3 ± 73.9 (195.8, 247.9)	**<0.001**	1.84
Inflammatory markers ‡				
IL-6 (pg/mL)	1.50 ± 0.41 (1.35, 1.66)	1.50 ± 0.36 (1.37, 1.63)	0.937	0.02
CRP (mg/L)	1.65 ± 2.05 (1.05, 2.57)	2.49 ± 2.23 (1.81, 3.41)	0.073	0.04
Immunological Markers ‡				
Total Leukocytes (cells/uL)	6316.3 ± 1441.2 (5821.1, 6853.7)	6686.5 ± 1370.6 (6221.0, 7186.8)	0.297	0.26
Total Lymphocytes (cells/uL)	2473.8 ± 660.2 (2248.5, 2721.7)	2371.2 ± 692.8 (2139.5, 2628.1)	0.579	0.15
CD3+ T Lymphocytes (cells/uL)	1828.7 ± 525.5 (1650.0, 2026.8)	1791.1 ± 559.3 (1604.7, 1999.2)	0.797	0.07
CD4+ T Lymphocytes (cells/uL)	1006.6 ± 374.6 (881.2, 1149.9)	960.2 ± 327.4 (851.6, 1149.9)	0.627	0.13
CD8+ T Lymphocytes (cells/uL)	732.3 ± 251.4 (647.6, 828.0)	698.6 ± 234.9 (620.6, 786.3)	0.594	0.14

WBLH, whole body less head; CTX, C-terminal telopeptide; IGF-1, insulin-like growth factor 1; CRP, C-reactive protein. #Model adjusted for sex and age from PHV (MO) and LST. ^‡^Model adjusted by sex, age from PHV (MO), and fat mass (g). *One missing data in the non-sport group.

Significant results are presented in bold.

Finally, the mediation analyses presented in **Table 3** were performed to confirm the absence of a mediating effect of inflammatory markers on the association between training load and aBMD among swimmers. In the mediation models of IL-6, a direct effect was found between training load and aBMD in the lower limbs (β=-0.153, p=0.025) and TBLH (β=-0.098, p=0.034), while no indirect effect of IL-6 was found (p>0.05). The mediation models of CRP showed no indirect effect (p>0.05), while the training load directly affected aBMD in the upper limbs (β=-0.013, p=0.019), lower limbs (β=-0.024, p=0.009), total spine (β=-0.015, p=0.034), and TBLH (β=-0.017, p=0.005).

**Table 3:** Mediation models of the association between training load and aBMD by inflammatory markers (IL-6 and CRP).

IL-6	Total effect	Controlled direct effect	Reference interaction	Mediated Interaction	Pure indirect effect
	*β* (95%*CI*)	*β* (95%*CI*)	*β* (95%*CI*)	*β* (95%*CI*)	*β* (95%*CI*)
aBMD					
Upper limbs	-0.0386 (-0.1507 to 0.0735)	-0.0650 (-0.1444 to 0.0143)	-0.0259 (0.1373 to 0.0854)	0.0259 (-0.0321 to 0.0839)	0.0264 (-0.0342 to 0.0871)
Lower limbs	-0.0855 (-0.3534 to 0.1824)	**-0.1533** **(-0.2875 to -0.0191)**	-0.0636 (-0.3280 to 0.2009)	0.0636 (-0.0616 to 0.1888)	0.0678 (-0.0661 to 0.2017)
Spine	-0.0491 (-0.1996 to 0.1013)	-0.0858 (-0.1838 to 0.0122)	-0.0350 (-0.1839 to 0.1139)	0.0350 (-0.0405 to 0.1106)	0.0367 (-0.0434 to 0.1167)
TBLH	-0.0559 (-0.2234 to 0.1115)	**-0.0978** **(-0.1880 to -0.0076)**	-0.0396 (-0.2051 to 0.1260)	0.0396 (-0.0400 to 0.1192)	0.0419 (0.4289 to 0.1266)

CRP	Total effect	Controlled direct effect	Reference interaction	Mediated Interaction	Pure indirect effect
	*β* (95%*CI*)	*β* (95%*CI*)	*β* (95%*CI*)	*β* (95%*CI*)	*β* (95%*CI*)
aBMD					
Upper limbs	**-0.0130** **(-0.0244 to -0.0016)**	**-0.0133** **(-0.0246 to -0.0021)**	0.00008 (-0.0013 to 0.0014)	-0.00008 (-0.0007 to 0.0005)	0.0003 (-0.0017 to 0.0022)
Lower limbs	**-0.0227** **(0.0421 to -0.0033)**	**-0.0238** **(-0.0417 to -0.0060)**	0.00010 (-0.0015 to 0.0017)	-0.0010 (-0.0009 to 0.0007)	0.0011 (-0.0063 to 0.0086)
Spine	**-0.0146** **(-0.0287 to -0.0006)**	**-0.0146** **(-0.0287 to -0.0006)**	0.0003 (-0.0041 to 0.0047)	-0.0003 (-0.0022 to 0.0016)	-0.00005 (-0.0009 to 0.0008)
TBLH	**-0.0168** **(-0.0298 to -0.0039)**	**-0.0174** **(-0.0296 to -0.0053)**	0.0001 (-0.0017 to 0.0019)	-0.0001 (-0.0009 to 0.0007)	0.0006 (0.0036 to 0.0048)

Adjusted by chronological age and age at peak height velocity (PHV). TBLH, total-body less head. Analysis were carried with swimmers only (n=30).

Significant results are presented in bold.

## 4 Discussion

This cross-sectional study aimed to analyse whether adolescent swimmers present different concentration patterns of inflammatory, immunological, anabolic, and bone markers compared with adolescents who do not practice organised sports and whether inflammatory variables mediate the association between training load and aBMD in adolescent swimmers. The main findings showed that although swimmers present lower BMD values in most body regions, they have higher concentrations of calcium, 25-hydroxy vitamin D, and IGF-1. Finally, in addition to the groups showing similar values for bone metabolism, immunological markers, and inflammatory variables, these inflammatory markers did not mediate the negative association between training load and BMD in swimmers.

The literature consistently states that swimming does not appear to be an effective sport for improving BMD (4), and our findings corroborate this statement. However, while some studies have shown a neutral effect of sports compared to controls (24) or a gain but with lower magnitude (25), others have reported a considerable negative effect, (5, 26) including the present study, where swimmers presented lower BMD compared to the non-sports group, except for in the upper limbs. To support this statement, a recent meta-analyses (27), demonstrated that in human models, swimming can present a neutral or negative effect on bone depending to anatomical region. The negative effect seems to occurs mainly in studies that include swimmers with a higher frequency of weekly training and consequently, a higher training load. Therefore, it was speculated that the physiological and inflammatory consequences of training could partially explain this phenomena, in addition to the environment without mechanical stimulation. Nonetheless, our results do not confirm these hypotheses and reinforce the fact that swimmers have lower aBMD, especially in the hypogravitational environment (4).

Competitive swimmers have a higher training load and consequently, higher daily training volume (i.e., time in water) (2). Therefore, the longer the time in the water (hypogravitational environment), the shorter the daily duration of mechanical stimulation from weight-bearing activities on land, even those not directly linked to sports practice. Consequently, less stimulation of bone tissue and lower BMD were observed in adolescents who did not practice sports. This result is interesting because it reinforces the idea that despite the high muscle activity during swimming, which is a significant predictor of BMD (28, 29), these muscle contractions do not seem to be effective in bone tissue, mainly in the lower limbs and whole body. In fact, studies have found that the relationship between the muscle and bone unit is different in swimmers (24), because the sport does not involve eccentric muscle contractions, in addition to low strain magnitude/strain during practice (30–32). Thus, dryland training should be recommended for swimmers, which can assist performance and is beneficial for the accumulation of bone mass (4, 33).

Furthermore, calcium, vitamin D, and IGF-1 were the only blood variables that showed concentration differences in the swimmers. Young athletes at a competitive level usually receive nutritional monitoring of calcium and vitamin D to improve the athlete’s health performance and avoid growth and developmental delays (34). Therefore, although we did not control for food intake, a diet rich in these nutrients justifies these results (34). In contrast, high IGF-1 concentrations appear to be associated with swimming practice. The concentrations of peptide and protein hormones, such as IGF-1, increase after physical exercise (35, 36); therefore, it is expected that swimmers will have higher concentrations of these hormones. However, despite its anabolic effect on different tissues such as muscle and bone, swimmers had lower BMD.

Despite the innovative aspects of the study, such as comparisons of different blood markers and testing the possible mediating effect of inflammatory markers, some limitations need to be acknowledged. First, the cross-sectional design prevented cause-and-effect interpretations. Second, the study did not analyse the food intake of the participants, which could indirectly affect any of the analysed markers. In addition, the sample size did not allow for analysis stratified by sex and age groups. Future studies are encouraged to explore the association between training load and BMD, as well as its potential mediators, in adolescents of different age groups (e.g., 10–14 and 15–19 years), stratified by sex and considering athletes with higher levels of sport engagement. The IL-6 assessment also had limitations, as the kit sensitivity did not detect values ​​below 1.5. Lastly, the method used to estimate the training load was subjective, and therefore presents greater limitations compared to objective methods previously applied in studies involving swimmers (37).

In summary, although adolescent swimmers at the competitive level had lower BMD in most body segments than those in the non-sports group, they did not show differences in bone turnover, inflammatory, or immunological markers. In contrast, swimmers presented with higher concentrations of IGF-1, calcium, and 25-hydroxy vitamin D. Furthermore, although the training load was negatively associated with the aBMD, IL-6 and CRP levels did not mediate this association. These results have two possible explanations. First, other biological markers need to be checked for an association between the training load and bone to confirm the findings. In contrast, this could reinforce the hypothesis that swimmers have lower aBMD, especially due to the hypogravitational environment, and should be encouraged to include dryland training in their training routines.

## Data Availability

The raw data supporting the conclusions of this manuscript will be made available by the authors, without undue reservation, to any qualified researcher.
