# Correction: Inflammatory markers as potential mediators on the negative association between training load and bone mineral density in adolescent competitive swimmers: ABCD-growth study

**DOI:** 10.3389/fendo.2025.1686139

**Published:** 2025-09-11

**Authors:** Ricardo R. Agostinete, Pedro H. Narciso, Manuel João Coelho-e-Silva, Renata M. Bielemann, Luis Alberto Gobbo, Bruna Camilo Turi-Lynch, Romulo Araújo Fernandes, Dimitris Vlachopoulos

**Affiliations:** ^1^ Laboratory of Investigation in Exercise (LIVE), Department of Physical Education, Sao Paulo State University (UNESP), Presidente Prudente, Brazil; ^2^ CIDAF (UID/DTP/04213/2016), Faculty of Sport Sciences and Physical Education, University of Coimbra, Coimbra, Portugal; ^3^ Post-Graduate Program in Nutrition and Foods, Federal University of Pelotas, Pelotas, Brazil. Post-Graduate Program in Epidemiology, Federal University of Pelotas, Pelotas, Brazil; ^4^ Skeletal Muscle Assessment Laboratory (LABSIM), Department of Physical Education, School of Technology and Sciences, São Paulo State University (UNESP), Presidente Prudente, Brazil; ^5^ Department of Physical Education and Exercise Science, Lander University, Greenwood, SC, United States; ^6^ Children’s Health and Exercise Research Centre, Sport and Health Sciences, University of Exeter, Exeter, United Kingdom

**Keywords:** youth, athletes, bone health, cytokines, hormones

The information “This study was financed in part by PROPG/UNESP through call ${\text{n}}^{\underline{\text{o}}}$ 00/2025” was erroneously omitted from the **Funding** statement in the original manuscript. The original text said “The author(s) declare that financial support was received for the research and/or publication of this article. This study was supported by the São Paulo Research Foundation-FAPESP (2017/09182-5, 2018/24164-6, and 2015/19710-3). Pedro Henrique Narciso received a grant from the FAPESP (2022/08875-5).”

The corrected **Funding** statement is presented below.

“The author(s) declare that financial support was received for the research and/or publication of this article. This study was supported by the São Paulo Research Foundation-FAPESP (2017/09182-5, 2018/24164-6, and 2015/19710-3). Pedro Henrique Narciso received a grant from the FAPESP (2022/08875-5). This study was financed in part by PROPG/UNESP through call ${\text{n}}^{\underline{\text{o}}}$ 00/2025.”

The original version of this article has been updated.

